# Supplementary material for: Working conditions of the clinical health workforce in the public health facilities in Bangladesh
Source: PLoS One. 2023 Nov 20;18(11):e0294224. doi: 10.1371/journal.pone.0294224 (PMC10659196; doi:10.1371/journal.pone.0294224)
Supplement: Attachment — Submitted filename: Response to Reviewers.docx [file pone.0294224.s002.docx]

Humayun Kabir

Academic Editor

PLOS ONE

Subject: Rebuttal Letter and Revised Manuscript Submission

Dear Dr. Kabir,

We would like to express our sincere gratitude to you and the reviewers for their valuable time and insightful feedback on our manuscript titled **Working conditions of the clinical health workforce in the public health facilities in Bangladesh**. We appreciate the constructive comments and suggestions, which have greatly contributed to the improvement of our study. In response to the reviewers' comments, we have carefully revised the manuscript, and we submit the revised version along with this rebuttal letter.

**Reviewer 1:**

Comment: Overall, the English is good, although there is some overuse of superlatives and the occasional quaint expression. This is not affecting the meaning but could readily be addressed by some editing. e.g. “Comprehensively measured scientific evidence on the level of working conditions of the health workforce is highly imperative for appealing effective policy interventions”

Response: We appreciate your feedback and constructive comments on our manuscript. We have carefully reviewed your suggestion regarding the overuse of superlatives and quaint expressions in our manuscript, and we agree with your observation. In response to your comment, we have thoroughly revised the manuscript to address this issue. We have carefully examined the text and made appropriate edits to reduce the overuse of superlatives and eliminate any quaint expressions that may have been present. Specifically, we have revised the sentence you mentioned as an example ("Comprehensively measured scientific evidence on the level of working conditions of the health workforce is highly imperative for appealing effective policy interventions") to ensure a more concise and straightforward expression of the intended meaning.

Comment: There is a quite an elaborate sampling strategy described in the methods but very little is made of this in the results. I wondered whether how the sampling strategy earns its place in the paper when so little is made of it in terms of the analysis and reporting?

Response: We have carefully considered your comment regarding the elaboration of the sampling strategy in the methods section and its limited representation in the results section. We agree that the sampling strategy plays a crucial role in ensuring the validity and generalizability of our findings, and it is important to properly address this aspect in the manuscript. To address this concern, we have revised the results section to provide a more comprehensive explanation of how the sampling strategy influenced our analysis and reporting. Specifically, we have included additional information on how the multi-stage sampling technique was implemented, including the selection of districts and sub-districts, and the random selection of clinical workforces within those areas. We have also described the sample size and composition in more detail. Furthermore, we have expanded upon the implications of the sampling strategy in the interpretation of our results. We now discuss how the selected sample is representative of the target population and acknowledge the limitations and generalizability of our findings based on the specific sampling approach.

Comment: Was there a theory to justify your sampling strategy?

Response: We appreciate your comment and feedback regarding the sampling strategy of the study. The study's sampling strategy is based on a combination of practical considerations and statistical principles. The multi-stage sampling technique was employed to ensure that the sample is representative of the clinical health workforce in the selected districts and upazilas of Bangladesh. The selection of the study area was based on the greater administrative divisions of Bangladesh, which provides a diverse range of geographic and administrative areas. While there was no specific theory used to justify the sampling strategy, the study employed a systematic sampling method to ensure the representativeness of the sample, which is consistent with statistical principles. The systematic sampling method involved using a list of all clinical staff in the selected healthcare facilities for the randomization of the respondents. Overall, the sampling strategy was designed to ensure that the sample is representative of the clinical health workforce in the selected districts and upazilas of Bangladesh and to maximize the generalizability of the study findings.

Comment: Were there differences between the four districts and eight upazilas?

Response: Yes, upazilas means sub-districts which are the second lower tier of the administrative structure in Bangladesh whereas the districts are the third lower administrative unit. Around 0.6-1.5 million people live in upazila whereas on an average nearly 4.5 million people live in districts. There are also differences in the working conditions status across the upazila and district.

Comment: Who is in the study (i.e. the participants) is usually provided in the results not the methods. The following description of participants should be moved the results, “The study includes 45 physicians and 123 other clinical staff from the UHCs, and 64 physicians and 87 other clinical staff from the DHs using a random sampling technique. Thus, we interview a total of 319 clinical staff (physicians: 109, other clinical staff: 210).

Response: We agree that providing information about the participants is more appropriate in the results section, as it directly relates to the findings of the study. To address this concern, we have revised the manuscript as per your suggestion. We have relocated the description of participants from the methods section to the results section. In the revised version, we now provide the following information in the results section:

"The study included a total of 319 clinical staff, comprising 109 physicians and 210 other clinical staff. Specifically, we interviewed 45 physicians and 123 other clinical staff from the UHCs, and 64 physicians and 87 other clinical staff from the DHs.”

Comment: The paper describes how they dealt with neutral in a Likert-type response. “Following the literature, the study considers the neutral option along with disagreeing and strongly disagree options for explaining poor working conditions because people often select the neutral option to respond indifferently or not positively (15,16). Refs 15 and 16 are papers written by the first author and the first refence only states much the same thing as is stated in the paper under review, without any referencing to support their statement “We consider the neutral option along with disagreeing and strongly disagree options for defining negative perception. The reason is that usually, an individual in Bangladesh chooses the neutral option if she likes to respond indifferently or not positively.” The second paper does not even refer to neutral responses. The second reference (that is number 16) should certainly be removed. Reference #15 provides no more no support for the assertion other than that they have used this approach before. It is important that authors describe how they have dealt with the data but they should not dress it up as it being based upon evidence which if it does exist is not provided.

Response: We have carefully reviewed your comments and made the necessary revisions to address this concern. In response to your comment, we have removed both references (Reference #15,16) that did not provide relevant support to our statement. To ensure transparency and accuracy in our reporting, we have revised the corresponding section of the manuscript as follows: "The study reports the percentage of respondents who agreed or strongly agreed with the statements related to good working conditions. For poor working conditions, the study merges the neutral option with the ‘disagree’, and ‘strongly disagree’ options. This is done to account for respondents who may have selected the neutral option due to indifference or lack of positivity toward the statement in question."

Comment: The Results should open with the overall description of the respondents (moved from the methods above).

Response: We have revised the manuscript accordingly. We have revised the corresponding section of the manuscript as follows “The study interviews a total of 319 clinical health workforce, with 45 physicians and 123 other clinical staff from the UHCs, and 64 physicians and 87 other clinical staff from the DHs.”.

Comment: It is not wrong but clunky saying “significant (p-value ≤.05)” each time – conventionally, these things are reported just as (p≤0.05)

Response: In response to your comment regarding the reporting of statistical significance, we agree that the phrase "significant (p-value ≤.05)" can be clunky and unconventional. We have carefully reviewed and revised our manuscript accordingly. We now consistently report statistical significance as "(p ≤.05)" throughout the text.

Comment: Most people aged 40 years or above would not be happy to be called elderly. Suggest change “elderly” to “older” throughout.

Response: We have revised the corresponding section accordingly.

Comment: The authors report that they did consult the literature in developing their list of various working conditions. Almost all of the factors were considered important by the respondents. Being paid on time and the relationship with co-workers were reported as the most important determinants for a good working environment. However, it may be considered is a weakness in a study design to just provide a list without requiring any process of deciding the relative importance of them i.e. ranking their importance, rather than that are very important.

Response: Regarding your comment about the weakness of our study design in not requiring respondents to rank the importance of the various working conditions, we agree that this could have been a valuable addition to our methodology. In observation, we recognize that ranking the importance of the factors would have provided more nuanced and informative results. In future studies, we will consider incorporating this approach to our methodology.

However, our additional explanation is that while the questionnaire did not explicitly require respondents to rank the importance of each component, the study did employ a 100-point scale to assess and rank the perceived importance of the components of working conditions by the clinical workforces in delivering quality healthcare. With this the study found that all the indicators were perceived as very vital for delivering quality healthcare, with some indicators being ranked higher than others in terms of importance. Still, considering your comment we did some modification in our method and result section adding rank of the components based on their mean weight score in Table 3. In result, we also explained the ranking of the components of working conditions which are derived from mean weight score based on their relative importance to delivering quality care.

In result section we have explained as follows “In terms of importance, receiving monthly salary in due time is top-ranked (99.15) to provide quality healthcare, followed by consistent availability of medicines and MSR (98.04), adequate mentoring and support to perform duties (97.57), good relations with co-workers (97.50), uninterrupted power supply (97.41), and so on.”

Comment: The authors state that the magnitude of working conditions is comparatively poor for men younger workforce and some others. However, it is not clear whether the conditions are different for them or whether the men are simply are more critical of the conditions. Perhaps men for example have higher expectations then women? it is also interesting that women felt they were not being able to access the training that they need and again it should be discussed in whether there is a gender bias in what training is available for women or whether women just feel they need additional training in order to feel competent in delivering care. These possibilities should be discussed.

Response: This is very insightful comment. We have discussed all these possibilities in discussion section. We have revised the corresponding section as follows “Men may have higher expectations of working conditions than women. However, the study does not find any evidence to suggest that men and women have different expectations of working conditions. The study also highlights that women do not receive the required training, which could be due to a lack of training opportunities available for women or a result of gender bias in the provision of training. We believe that it is important to further investigate these issues to ensure that all clinical staff, regardless of gender, have equal access to training opportunities to improve the quality of care they provide.”

**Reviewer 2:**

Comment: There is no clear objective of this study, merely to trigger policy discussion? Improving efficiency and effectiveness of the healthcare service delivery system is beyond the scope of this paper.

Response: We appreciate your valuable feedback and comments on our manuscript. Your insights have been instrumental in refining the focus and objectives of our study. We agree that the initial version of the manuscript did not clearly state the objective of the study. We have revised the introduction and methodology sections to provide a clear and concise objective for our research. We have revised the objective in the abstract section as follows "Therefore, the study aims to assess the level of working conditions of the clinical health workers in Bangladesh and their relative importance in delivering quality healthcare services." Our aim is to contribute empirical evidence to inform policy discussions and potential interventions aimed at improving the working conditions of healthcare professionals. However, we would like to clarify that while our study aims to shed light on the current state of working conditions, we acknowledge that improving the efficiency and effectiveness of the healthcare service delivery system is a broader issue that extends beyond the scope of this paper. Our focus is primarily on evaluating the working conditions themselves and providing valuable insights to inform policy considerations in this specific area.

Comment: The score (weighted mean score) of 3.4 (how? and why?) was considered to represent poor working conditions?

Response: We have calculated the weighted mean score by following method “we compute a weighted mean score that accounts for the weight given by the clinical workforces to each components, as well as their reported score for that components.” We have considered a mean score of 3.4 as poor working condition because in method we have determined 75^th^ percentile as a cut-off point to categorize the working conditions as either good or poor, based on our understanding of the issue and the study’s context. This is also supported by previous literature (reference #26 in our manuscript). Therefore, we have revised the corresponding section in data analysis part as follows “To determine the overall status of the working conditions, the study computes a weighted mean score that accounts for the weight given by the clinical workforces to each components, as well as their reported score for that components. We use the 75^th^ percentile as a cut-off point to categorize the working conditions as either good or poor, based on our understanding of the issue and the study’s context”.

Comment: The average (mean?) weight score (out of 100 points) indicating that all parameters included were pre-requisites for delivering effective and efficient services??? This is very confusing and contradictory.

Response: Upon careful reconsideration, we agree that the statement regarding the average weight score (out of 100 points) and its interpretation as prerequisites for delivering effective and efficient services may have been misleading. In the revised manuscript, we have provided a clearer and more accurate description of the average weight score. The weight score represents the perceived importance or significance assigned to each parameter by the participants on a scale of 0 to 100. We have refrained from making claims about these parameters being prerequisites for effective and efficient service delivery, as it requires a more nuanced understanding and comprehensive analysis beyond the scope of our study.

Comment: The conclusion section did not reflect the actual work done in this research.

Response: Your comment regarding the discrepancy between the conclusion section and the actual work conducted in this research has been carefully considered, and we have made the necessary revisions to address this concern. Upon review, we acknowledge that the initial conclusion section did not accurately reflect the findings and contributions of our research. In the revised manuscript, we have extensively revised the conclusion section to align it more closely with the actual work conducted in our research. We have summarized the key findings, highlighted the implications of our study, and discussed the relevance of our results in the context of the existing literature.

Comment: Introduction section is lacking the literature related to work place and working condition in Bangladesh and other developing countries and the reflections on quality of services and staff.

Response: In response to your comment, we have thoroughly revised the introduction section to include a comprehensive review of the existing literature related to workplace and working conditions in Bangladesh and other developing countries. We have incorporated relevant studies that discuss the impact of working conditions on the quality of services and the well-being of staff. By doing so, we aim to provide a robust background and theoretical framework for our study, highlighting its relevance and contribution to the existing body of knowledge.

Comment: Working condition and how significant in terms of efficiency, effectiveness and satisfaction of the health care team.
Response: We have discussed these things in introduction section of our manuscript as follows “Work commitment is vital, especially for the healthcare workforce. Studies show that different components of working condition play an important role to determine employees' commitment to work which is associated with the provision, quality, accessibility, and affordability of healthcare (ref#16). Thus, working conditions affect the efficiency and effectiveness of the healthcare team. Health workers who work in an environment with inadequate resources and equipment may face delays in providing care, which can compromise patient outcomes (ref#17). Adequate staffing levels, equipment, and supplies are necessary for efficient healthcare delivery (ref#18). Moreover, workload and job stress can affect the efficiency of health workers, leading to burnout and turnover”.

Comment: What kind of strategy adopted and lunched by the government and public sectors to improve the work conditions in Bangladesh and other developing countries with similar circumstances?

Response: In response to this comment, we have discussed the thing in introduction section of our manuscript as follows “The government of Bangladesh has adopted various strategies to improve working conditions in healthcare facilities including construction of new healthcare facilities and the renovation of existing ones, and recruiting and training more healthcare workers (ref#4).”

Comment: “Although a growing body of the literature focuses on defining the components of working conditions of the health workforce, there is no scientific research globally on the status of working conditions measured comprehensively” this is not correct and needs references.

Response: In response to your feedback, we have carefully revised the sentence. We have now provided a more accurate and evidence-based statement regarding the existing literature on the comprehensive measurement of working conditions globally. However, by this sentence what we have meant is that there are studies in defining and identifying various components of working conditions of health workforce. But study on assessing the existing or perceived level of working conditions of health workforce is limited. However, we have revised the corresponding section in introduction part of our manuscript as follows “There is limited research on measuring the status of working conditions of clinical health workers in both national and international context. As evidenced above, some studies have focused on specific aspects of health workers' working conditions in Bangladesh, such as job satisfaction or motivation. However, a comprehensive analysis of the various dimensions of health workers' working conditions is lacking.”

Comment: There is no concrete rationale for the study.

Response: In response to your comment, we have revised the introduction section to include a more explicit and detailed rationale for conducting the study. We have incorporated relevant literature and highlighted the gaps in knowledge that our research aims to address. However, we have revised the corresponding section as follows “There is limited research on measuring the status of working conditions of clinical health workers in both national and international context. As evidenced above, some studies have focused on specific aspects of health workers' working conditions in Bangladesh, such as job satisfaction or motivation. However, a comprehensive analysis of the various dimensions of health workers' working conditions is lacking. Such an analysis is critical in understanding the challenges and opportunities in improving the working conditions and consequently, the quality of care provided to the population. The current study aims to fill this knowledge gap by providing a comprehensive analysis of the working conditions of clinical health workforce in public health facilities in Bangladesh.”.

Comment: Line 88 to line 90: remove

Response: Removed and revised accordingly.

Comment: Replace data with sampling methods

Response: Replaced accordingly.

Comment: What was your study design?

Response: Cross-sectional study design. However, we have revised the corresponding section in the method as follows “The study follows a cross-sectional study design and analyses the primary data collected from January to March 2022 applying a quantitative method.”

Comment: Setting should be mentioned (what is upazilas? the second lower tire of administrative structure in Bangladesh) should be defined, and how many each can serve.
Response: We have revised the manuscript accordingly. However, Upazilas means sub-districts which are the second lower tier of the administrative structure in Bangladesh whereas the districts are the third lower administrative unit. Around 0.6-1.5 million people live in upazila whereas on an average nearly 4.5 million people live in districts.

Comment: Your sampling methods should be proposed as a Multi-stage sampling methods with each stage should be provided in details: first how many districts were included in the primary selection (4 districts were randomly selected out of ----) for the secondary care centers and 8 upazilas (randomly selected from ------) form the greater administrative divisions to represent the primary care facilities.

Response: Thank you for your valuable feedback. We have revised the manuscript accordingly. However, we have stated this in our method as follows “ We adopt a multi-stage sampling technique to select a representative sample of the clinical health workforce. The study selects four districts out of 64 and eight upazilas (the second lower tier of the administrative structure) out of 495 from the four greater administrative divisions (Dhaka, Chattogram, Rajshahi, and Khulna) of Bangladesh as the study area. In the first stage, the study randomly selects four districts drawing one from each of the four administrative divisions. In the second stage, the study selects a total of eight upazilas, taking two from each of the selected districts using the same technique. In the third stage, the study employs a random sampling technique to select clinical workforce from the both District Hospital (DH) and Upazila Health Complex (UHC) of the selected districts and upazilas.”

Comment: The second stage is the selection of interviewees (systematic sampling method should be followed, database or list of all clinical staff in the selected centers should be available for that purpose).

Response: We followed the process in reality, but did not report in previous version of our manuscript. However, we have revised the method section addressing your comment. We have incorporated the following sentences in our revised manuscript “To ensure the representativeness of the sample, the study utilizes a systematic sampling method, which involves using a list of all clinical staff in the selected healthcare facilities for the randomization of the respondents. At the outset, we contact 350 clinical workforces for interviews, consisting of 120 physicians and 230 other clinical staff, to serve as an indicative sample. Regrettably, 31 of them decline to participate. Consequently, we conduct interviews with 319 clinical workforces, comprising 109 physicians and 210 other clinical staff.”

Comment: Sample size should be determined before selection of participants (not found).

Response: We did it in reality, but missed to report it in our previous version. We have addressed it in following manner “At the outset, we contact 350 clinical workforces for interviews, consisting of 120 physicians and 230 other clinical staff, to serve as an indicative sample. Regrettably, 31 of them decline to participate. Consequently, we conduct interviews with 319 clinical workforces, comprising 109 physicians and 210 other clinical staff.”

Comment: Based on literature review you had consulted, what were the pros and cons for each definition of working conditions, how was each instrument reliable and valid?

Response: We pretested the questionnaire for checking reliability and validity. We also conducted psychometric analysis to assess the validity, and reliability of the components.

Comment: Line 117: not clear

Response: We have revised the sentence as follows “The questionnaire contains separate statements for each component of working conditions for examining the perceived status of the working conditions of the clinical workforces as stated in Table 1.”

Comment: How did you invite participants? was it a personal interview and by whom? And in what language the data collection form was administered?
Response: For participant recruitment, we used a multi-stage sampling technique. We randomly selected 319 clinical workforces from four districts and eight sub-districts in Bangladesh. The selection process involved identifying primary and secondary-level healthcare facilities and then randomly selecting participants from those facilities. Regarding the data collection method, we conducted personal interviews with the participants. The interviews were administered by trained research personnel who were familiar with the study objectives and questionnaire. These individuals were experienced in conducting interviews and followed standardized procedures to ensure consistency and reliability in data collection. The data collection form was administered in the local language, Bangla. We chose to use the participants' native language to ensure better understanding and accurate responses. The questionnaire was translated into Bangla by a team of bilingual experts and pretested for clarity and comprehension before the actual data collection.
 Comment: How many responded to your invitation? how many refused/declined? were they different form the respondents?

Response: A total of 319 responded out of 350 invitations. 31 declined to participate. Yes, they were different from the respondents. However, we have revised it as follows “At the outset, we contact 350 clinical workforces for interviews, consisting of 120 physicians and 230 other clinical staff, to serve as an indicative sample. Regrettably, 31 of them decline to participate. Consequently, we conduct interviews with 319 clinical workforces, comprising 109 physicians and 210 other clinical staff.”

Comment: Table 1; Item 1: subjective

Response: The is a subjective statement. But we think it is okay because we are examining self-reported/ perceived status of working conditions by the clinical workforces.

Comment: Table 1: Item 2: enough staff?? How many is the ideal?

Response: As we are examining the perceived status of working conditions, no specific number is need for making it ideal. We just want assess that what respondents perceive that the existing number of staff in his/her respective facilities is enough or not.

Comment: Item 3: roles vs. responsibility (double barrel question)

Response: We know double barrel question can be problematic because it does not allow for a clear and distinct answer to each issue. But our stand is that we asked the respondents to answer (choose scale) keeping on mind the both issues (on an average rating).

Comment: Item 5: define efficiency
 Response: In our study work efficiency meant as not wastage of time for delivering services to the patients.

Comment: I suggest to categorize these items into fewer dimensions: work performance indicators (volume, training, equipment, and staffing), psychological (rewarding, protection, recognition, burn out and etc., ), and physical working conditions (layout, color and lighting, water, sanitation etc.,)

Response: We do not want to categorize the items into fewer dimensions for the following reasons. Firstly, we selected the original categorization to distinguish between different aspects of the working conditions that are important to consider separately. Combining them into broader categories may result in a loss of information and complexity, which could affect the accuracy and validity of the findings. Secondly, as data have already been collected and analyzed, changing the categorization requires re-analyzing the data. It could also affect the comparability of the results with previous studies. Thirdly, the original categorization is based on a solid literature review, which justifies the relevance and importance of each item. Combining them into broader categories could result in a less rigorous analysis. Finally, it is more transparent to report the results according to the original categorization, as this allows readers to understand how the items were measured and how they contribute to the overall analysis.

Comment: How the questionnaire customized?

Response: The questionnaire was customized in several ways to ensure its relevance and suitability for the study population and country context. First, we reviewed the existing literature on working conditions among clinical health workforces in Bangladesh and identified relevant constructs and variables to include in the questionnaire. Second, we piloted the questionnaire with a small sample of clinical health workers to test its reliability and validity and made further revisions based on the pilot study results. Finally, we translated the questionnaire into Bengali, the local language spoken by the study participants, to ensure its accessibility and cultural appropriateness. Overall, the customization process aimed to ensure that the questionnaire was valid, reliable, and relevant to the study population and effectively captured the key constructs and variables of interest for the study.

Comment: “In addition, the study measures the same indicators on a scale of 100 to assess weight based on their relative importance to provide quality healthcare”. Why and how?

Response: The sentence has been modified as follows “In addition, the study employs a 100-point scale to assess the perceived importance of the aforementioned components of working conditions by the clinical workforces in delivering quality healthcare” for clarity of meaning. We did this to fulfill second objective of our study for making rank of the components based on their relative importance for delivering quality care. We collected this information by directly asking the respondents by the trained enumerators.

Comment: Replace estimates with calculates (considered the tense)

Response: Replaced accordingly.

Comment: This section is completely not clear “The study calculates a weighted mean score adjusting the weight given by the respondents to each indicator with their reported score to calculate the overall status of the working conditions.

Response: This section is rewritten as follows for clarity and understandability “ To determine the overall status of the working conditions, the study computes a weighted mean score that accounts for the weight given by the clinical workforces to each components, as well as their reported score for that components.”

Comment: There is should a valid method to determine this cut-off (using percentiles), We define the working conditions as good when the weighted mean score is 4 or above, and poor when it is below 4.

Response: Revised accordingly. Based on our knowledge on the issue and study context, we use the 75^th^ percentile as a cut-off point to categorize the working conditions as either good or poor, and it supported by literature.

Comment: Finally, we use the chi-square test to assess whether the responses of the different categories of respondents are significantly different or not. Chi-square for which variables?

Response: We have revised the statistical tests used to assess the significance of differences between the responses of different categories of respondents. Instead of using the chi-square test, we have used non-parametric tests such as the Wilcoxon rank-sum test and the Kruskal-Wallis test. Specifically, we used the Wilcoxon rank-sum test to compare the responses of two groups and the Kruskal-Wallis test to compare the responses of three or more groups. We thank the reviewer for their suggestion, which led us to reconsider our statistical analysis and improve the rigor of our study. We have made the necessary changes to our manuscript to reflect these revisions.

Comment: This proposed questionnaire should be piloted on a sample beyond the selected candidates to measure reliability if any.

Response: The questionnaire had been piloted on a sample beyond the selected candidates to measure reliability. Considering your comment, we have revised the respective section in the method as follows “We pilot and customize the questionnaire to test its reliability and validity in the country context and made further revisions based on the pilot results.”

Comment: Construct validity should be verified using other method not the confirmatory factor analysis? And from line 155 to line 165: this section is totally incorrect and should be removed from the manuscript.

Response: We appreciate your feedback and would like to provide additional clarification on our choice of using confirmatory factor analysis (CFA). We agree that there are different methods available to assess construct validity, such as exploratory factor analysis (EFA), item response theory, and others. However, in our study, we chose to use CFA instead of EFA because we had a priori assumption about the underlying dimensionality of the construct. CFA allows us to test whether our set of indicators can explain a single latent construct called working conditions, as we hypothesized. In contrast, EFA is a data-driven approach that identifies the number and nature of underlying factors in the data, which may not align with our research question and hypothesis.

Furthermore, we acknowledge that there is no universal cutoff point for determining the significance of factor loadings in CFA. The literature reports different cutoff values ranging from 0.4 to 0.6, and some researchers recommend using modification indices and other fit indices to improve the model fit. In our study, we set the cutoff value as >0.5 based on previous studies in our field, but we also reported the factor loadings and fit indices to ensure the transparency and replicability of our analysis.

Finally, we understand that different methods exist to assess construct validity, and we appreciate your comment on our study. However, we believe that our use of CFA aligns with our research question and hypothesis and provides valuable information on the validity of our survey instrument.

 Comment: Table 2: should display both socio-demographics vs. the responses for each dimensions (and the relevant items).

Response: Thank you for your suggestion regarding Table 2. However, we respectfully decline to make the suggested changes. We believe that our current presentation of the socio-economic characteristics of the respondents provides valuable information and insight into the sample population. Additionally, we have presented the responses of different socio-economic groups on each dimension of working condition in other tables (Table 4, Table 5), which provide a comprehensive and clear picture of the relationship between socio-economic characteristics and the perceived importance of working conditions. We understand your concern regarding the need to display both socio-demographics and responses for each dimension and item, but we feel that the current presentation is more succinct and clearer.

Comment: Table 3: should display the responses in relation to the type of services (primary vs. secondary)

Response: Thank you for your comments. While we appreciate your suggestion, we have decided not to present the results of Table 3 in relation to the type of services (primary vs. secondary) as it is not within the scope of our research question and objectives. Our study aimed to explore the perceived status of working conditions and their relative importance in delivering quality healthcare among the clinical workforce, regardless of the type of services they provide. Therefore, we believe that presenting the results of Table 3 in relation to the type of services may not be relevant to our research question. However, we have presented the results of Table 4, which shows the responses on different dimensions of working conditions in relation to the type of services, to provide additional information to the readers.

Comment: Non parametric tests of significance should be used to compare scores in any table displayed.

Response: We have taken note of your recommendation and used appropriate non-parametric tests. We have used the Wilcoxon rank-sum test to compare two groups, whereas the Kruskal-Wallis test has been used to compare three or more groups. Thank you for your valuable feedback.

Comment: This section is so redundant as many parts of the tables mentioned in text of this section.

Response: We appreciate your input and have carefully revised the result section to ensure that it is more concise and avoids unnecessary redundancy. We have edited the text only to highlight the most important findings from the tables and have made sure that any additional information provided is necessary for a clear understanding of the results. We hope that this revision meets your expectations.

Comment: Table 5 and 6 are very confusing.
 Response: We appreciate and acknowledge your feedback. We understand your concern regarding the clarity of Tables 5 and 6 in the result section. We agree that these tables contain a large amount of data and variables, which can make them appear overwhelming. However, we have carefully designed these tables to present our findings accurately and comprehensively while fulfilling our research objectives. We present the data this way so that this may achieve our objective of providing a detailed analysis of the relationship between different variables. However, Table 5 represents the differences in responses and their significance according to respondents' different socio-demographic conditions whereas, Table 6 presents differences in mean weight scores put on various components of working conditions by the respondents of different socio-economic classes.

Comment: The study calculates the mean weight score of each indicator to assess their relative importance to provide quality healthcare by the health workforces in the facility. We ask the respondents to put weight on a scale of 100 on each of the indicators of working conditions considering their relative importance to deliver quality healthcare (this section belongs to the methods section)?

Response: We have removed it from result section, and revised accordingly.

Comment: Discussion section:
- Start your discussion with providing the answer to your research question!!!!
- The authors mentioned many segments form the results section with repetitions
- Very lengthy discussion with redundancy

Response: We have completely revised the discussion section in response to your comment. To address your first point, we have now revised the discussion section to start by directly answering our research question. By doing so, we provide a clear and focused introduction to the key findings of our study and their implications. This change helps strengthen the discussion's overall structure and ensures that the main research question is addressed upfront. Furthermore, we have carefully reviewed the segments from the results section mentioned in the discussion. We have eliminated any unnecessary repetitions and streamlined the presentation of our results. This revision ensures that the discussion highlights the key findings and their significance without unnecessary redundancy.

Comment: Conclusion: There should be a separate section for study limitations which are many

Response: We have added a separate section for presenting limitations of the study.

Comment: “The findings of the study are not directly comparable, as there is a paucity of evidence assessing the status of working conditions compared with different types of healthcare facilities and providers in both national and global contexts” this is totally incorrect.

Response: Upon careful consideration, we agree that our previous statement regarding the paucity of evidence comparing working conditions across different types of healthcare facilities and providers was incorrect. We acknowledge that existing literature analyses working conditions in various healthcare settings at the national and global levels. To rectify this oversight, we have conducted an extensive review of the relevant literature and have incorporated appropriate references that compare the findings of our study with previous research. These additional references provide context and support for the comparability of our results and contribute to a more comprehensive and well-grounded discussion of our findings.

Comment: This section is also confusing and did not reflect the actual work done.

Response: We have extensively revised the conclusion section to avoid confusion to readers, and to reflect the actual work done.

Comment: References to different data collection instruments used in the literature were not mentioned

Response: We agree that including references to the data collection instruments used in previous studies adds value to our manuscript and enhances the transparency of our research methodology. We have conducted a thorough review of the relevant literature and identified the key studies that employed similar data collection instruments to assess working conditions in healthcare settings. In our revised manuscript, we have included references to these studies, specifically highlighting the data collection instruments.

**Reviewer 3:**

Comment: the authors need to provide evidence to support the statement on p.4, lines 83–84. To my knowledge, there exists comprehensive measures working conditions in the hea;th sector, e,g,, Maassen et al (2021) DOI: 10.1371/journal.pone.0247530; ILO (2017) ISBN 978-92-2-130533-0; Kristensen (2010) DOI: 10.1177/1403494809354437, etc. Particularly, Kristensen's COPSOQ tool has been adopted and implemented in many countries.

Response: Thank you for your comment regarding the comprehensive measures of working conditions in the health sector. We appreciate your feedback and would like to address this concern.

Upon further review and consideration, we agree that there are existing measures of working conditions in the health sector, but limited. We apologize for the oversight in our initial manuscript. In the revised version, we have incorporated references including that are suggested by you, to the relevant literature highlighting the availability of measures for assessing working conditions in the health sector. These references provide evidence of previous studies that have utilized comprehensive measures and contributed to the existing body of knowledge in this area. By including these references, we acknowledge the importance of previous research and recognize that our study builds upon existing measures and methodologies. This strengthens the scientific foundation of our work and ensures that readers have access to the relevant literature on comprehensive measures of working conditions.

We sincerely thank you for bringing this to our attention and helping us improve the quality and accuracy of our manuscript.

**Reviewer 4:**

Comment: The title: This appear incomplete considering that the study focused on two major premise (lines 84-86) "assessing the level of working conditions of the health workforce...study also assesses the relative importance of the different factors of working conditions." What factors are these? How these are related should be the burden of this study yet the title has not adequately captured it.

Response: Thank you for your feedback regarding the title of our study. We understand your concern regarding the need to capture the two major premises of our research: assessing the level of working conditions and the relative importance of different factors of working conditions. While we appreciate your suggestion to change the title, we would like to provide an explanation for our decision to retain the original title.

The current title of our study was carefully chosen to convey the main focus of our research succinctly. While it may not explicitly mention the assessment of factors of working conditions and their importance in delivering quality care, we believe that the content of the study adequately addresses this aspect. Throughout the paper, we extensively discuss and analyze various factors related to working conditions, including but not limited to remuneration, availability of resources, workplace safety, and staff support systems. We examine the relative importance of the factors to provide quality care. These factors impact the overall level of working conditions and subsequently influence the quality of services the health workforce provides. By assessing the level of working conditions comprehensively, we indirectly consider the relative importance of different factors.

While we acknowledge the value of explicitly mentioning the factors in the title, we believe that our current title accurately reflects the essence of our research and is in line with established academic conventions. However, we have considered your comment and revised the introduction and methodology sections to explain the factors under investigation and their relationship to the study's objectives.

Comment: The abstract needs to be well articulated for such an important study. The context healthcare in line 36 is missing. The objective of measuring level of working condition appears incomplete.

Response: We appreciate your suggestion to provide a more comprehensive and context-specific abstract that better captures the importance of the study in the healthcare sector. We have completely revised the abstract to address these concerns and provide a clearer overview of our research. In the revised abstract, we have included a more explicit reference to the healthcare context in which the study is conducted. We have also refined the objective statement to provide a complete understanding of our aim to measure the level of working conditions among the health workforce. Additionally, we have ensured that the abstract highlights the significance of our findings in terms of implications for healthcare quality. We believe that these revisions have significantly improved the articulation and clarity of the abstract, aligning it more effectively with the overall purpose and scope of the study.

Comment: The description of the data collection instrument is inadequate, line 42: "...administers a customized…", what is customized about the instrument?

Response: The term "customized" about the data collection instrument indicates that the instrument was specifically developed and tailored for our study. It means that we did not use an existing standardized instrument but rather designed a questionnaire specifically focused on the Bangladesh context, capturing the relevant aspects of working conditions in the health workforce. In our revised manuscript, we have provided a more detailed explanation of the customization process, highlighting the elements and domains included in the instrument to ensure its relevance and appropriateness for assessing working conditions in our study context. We hope this clarification provides a clearer understanding of the customization aspect.

Comment: The result stated in the abstract reflecting the overall weighted mean score of the working conditions is on a scale of 5 which reflects a poor working condition does not demonstrate such since nothing about the instrument measurement scale is mentioned in the methodology section. What is the scale of measures for variables in the study?

Response: In response to your comment, we have included a detailed description of the scale of measurement used for the variables related to working conditions. We have now clearly mentioned the scale of measurement in the methodology section. To assess the working conditions, we utilized a Likert-type scale ranging from 1 to 5, where 1 represents a poor working condition, and 5 represents a good working condition. This scale was used to capture the participants' perceptions and evaluations of various aspects related to their working conditions. Furthermore, to determine the cutoff point for assessing poor or good working conditions, we have set the 75th percentile as the threshold. Participants scoring below the 75th percentile were categorized as experiencing poor working conditions, while those scoring at or above the 75th percentile were considered to have good working conditions. This approach allows for a more nuanced understanding of the distribution of working conditions among the participants.

Comment: At a certain stage in the narrative in the introduction should be a presentation of the theoretical and conceptual framework underpinning of what constitute the workload that is to be measured and the antecedent factors relative the the outcomes and consequences in a theoretical clarification. This provides the scientific foundation of the argument in the study.

Response: Considering your suggestion we have incorporated the following theoretical framework in the introduction section. “The positive association of working conditions and job satisfaction is supported by the job demands-resources (JD-R) model which provides a useful theoretical and conceptual framework for understanding the factors that contribute to the workload and well-being of healthcare workers. Job demands include factors such as workload, time pressure, emotional demands, and role ambiguity, while job resources include social support, autonomy, feedback, and opportunities for learning and development (Ref#14,15). The JD-R model predicts that high job demands and low job resources can lead to negative outcomes such as burnout and turnover intentions, while high job resources can lead to positive outcomes such as work engagement and job satisfaction (ref#14).”

Comment: The methodology has some merits but lack coherence. Table 1, shows 26 elements of the working condition measured on a 5-response Likert scale. My concerns are why "neutral", how is neutral scored in relation to others to create a rating scale to measure each participant in the study? What is the final rating scale for this variable? From my judgment, the reference scale for this variable should be a maximum of 104 points representing heavy workload. The cut-off defining threshold to what constitute unacceptable level should be defined and justified.

Response: We have used the neutral option in our analysis to account for respondents who may have selected it due to indifference or lack of positivity toward the statement in question. The neutral option is scored as 3 on the 5-point Likert-type scale, which is the midpoint of the scale. We merged the neutral option with the 'disagree' and 'strongly disagree' options to create a rating scale that measures negative attitudes toward the statements related to poor working conditions. Specifically, we assigned a score of 1 to 'strongly disagree,' 2 to 'disagree,' and 3 to 'neutral.' In contrast, we assigned a score of 4 to 'agree' and 5 to 'strongly agree' to measure positive attitudes toward the statements related to good working conditions. Based on these scores, we calculated a weighted mean score for each component of the working conditions. Finally, we used the 75th percentile as a cut-off point to differentiate between good and poor working conditions. Therefore, the final rating scale for this variable ranges from 1 to 5, with 3 being the neutral score and scores below 4 indicating negative attitudes, while scores 4 and above indicate positive attitudes.

Comment: The data analysis requires adequate organization and analysis to provide clear answers for the research questions, validity for the findings and conclusion.

Response: We appreciate your comments and have considered them while revising the manuscript. We agree that the data analysis needs to be adequately organized and analyzed to provide clear answers to the research questions, ensure the validity of the findings, and draw sound conclusions. We have thoroughly revised the methods and results sections to address your concerns. We have provided a detailed description of the data analysis procedures, including the statistical tests used and their justifications. We have also reorganized the results section to present the findings in a clear and concise manner, with appropriate references to the relevant tables. We believe that these revisions have improved the quality of the manuscript and have addressed the concerns raised by you.

Comment: The presentation of frequency distribution of all items of the instrument without synthesis of the variables in the study makes it difficult to interpret the findings.

Response: We appreciate your comments regarding the presentation of the frequency distribution of all items of the instrument in our manuscript. While we appreciate your feedback, we would like to explain that we intentionally presented the frequency distribution of all items of the instrument in order to provide a comprehensive understanding of the responses of the participants. We believe that presenting the individual items allows readers to understand better the specific areas that participants may have identified as good or bad in their working conditions. However, we have taken your comments into consideration and provided a detailed discussion of the key results to ensure that readers fully understand the implications of our findings. Thank you for your valuable feedback, which has helped us to improve the clarity and organization of our manuscript.

Comment: Overall language editorial is extensively required.

Response: We have thoroughly addressed the language editorial concerns to enhance the clarity and readability of the paper. We have conducted comprehensive language editing to ensure the manuscript adheres to proper grammar, syntax, and academic writing conventions. We have also paid attention to eliminating any instances of excessive superlatives and quaint expressions, as highlighted by the reviewers. The revised manuscript now reflects a more concise and academic language style.

**Reviewer 5**

Comment: The manuscript is written poorly. The introduction part merely two paragraphs and that too out of context. No scientific process or methods have been followed for the development/adoption of the data collection tool. The statistical analysis is not appropriate as per the study objectives, the presentation of data is below average. The manuscript is in a shape which cannot be improved with reviewers comments.

Response: Thank you for sharing the reviewer's comments on our manuscript. We appreciate their time and effort in reviewing our work, even though their comments were critical.

In response to the reviewer's comment about the poor writing quality, we acknowledge that there may be areas where our manuscript could be improved. We apologize for any confusion caused by the two-paragraph introduction, and we have revised it to provide a more comprehensive and contextualized overview of our study. We have also made significant efforts to improve the clarity and organization of the manuscript as a whole. Regarding the comment about developing or adopting the data collection tool, we apologize for any misunderstanding. We have included a detailed description of the development and adaptation process of the data collection tool in the Methods section of the revised manuscript. We believe that this clarification addresses the reviewer's concern adequately.

Regarding the critique of the statistical analysis, we have carefully reviewed our study objectives and made necessary adjustments to ensure the appropriateness of the statistical methods employed. We have also considered the specific comments the reviewer provided and revised the statistical analysis accordingly. As for the presentation of data, we understand the importance of effectively conveying our findings. We have revised the presentation of data to enhance clarity and ensure that the results are presented more organized and understandable.

While we understand the reviewer's concerns, we respectfully disagree with their statement that the manuscript cannot be improved with reviewer comments. We have carefully considered all the reviewer comments and made substantial revisions to address their concerns. We are confident that these revisions have significantly enhanced the quality and clarity of our manuscript. We sincerely hope that the revisions we have made address the concerns raised by the reviewer. We believe that our research has important contributions to make to the field.

We sincerely hope that these revisions address the concerns raised by the reviewers. We are confident that the revised manuscript represents a significant improvement and contributes to the existing literature on working conditions of clinical health workforces. We once again express our appreciation for the opportunity to submit our work to PLOS ONE.

Thank you for considering our revised manuscript and rebuttal letter. We look forward to hearing from you regarding the final decision.

Sincerely,

Md. Ragaul Azim

Corresponding Author

Institute of Health Economics, University of Dhaka

Email: [azim.ihe@du.ac.bd](mailto:azim.ihe@du.ac.bd)
