# Supplementary material for: Working conditions of the clinical health workforce in the public health facilities in Bangladesh
Source: PLoS One. 2023 Nov 20;18(11):e0294224. doi: 10.1371/journal.pone.0294224 (PMC10659196; doi:10.1371/journal.pone.0294224)
Supplement: Attachment — Submitted filename: Rereview_Bangladesh workforce PLOS One.docx [file pone.0294224.s003.docx]

The authors have extensively revised their manuscript in response to the many comments that they received from reviewers. I think the manuscript is much improved by this.

I am generally happy with the responses to the issues that I raised. The paper is an original contribution and there is generally good detail in the paper with it presented in an intelligible fashion with appropriate ethical approval.

I am sorry to obtuse, and I have carefully read the authors’ responses to Reviewer 2. As a reader of the manuscript, it is still difficult to understand how the mean weight score is calculated - is this across all respondents to each individual item? This seems clearer in the authors’ response than the paper. Similarly, the application of the 75^th^ percentile cut off also lacks seems a construct where further justification or at least explanation of what it means in practice is warranted.

Overall, the English is good but there are occasional errors. This may have been missed in the initial review but the paper is curiously written in future tense, as if the authors are reporting a protocol rather than on a study that has been completed. In fact, it is not even really in future tense e.g. (in the abstract) “The study aims… (rather than aimed) and Descriptive statistics, and “”bivariate analysis are used to analyze the data”(rather than Descriptive statistics and bivariate analyses were used to analyze the data.” Perhaps it is because this manuscript is part of a larger study that still is underway and will be reported. This could be a discretionary revision as it does not undermine the scientific reporting although it is not following conventional reporting style.

The authors misunderstood my query as to whether there were differences between the four districts and eight upazilas. I meant with respect to the findings. However, I accept that in a paper of this size responding to this in terms of difference in the workforce across a large city like Dhaka may not be considered a priority.

Overall, I feel this paper is important and deserves to be published. More clarity /justification in the statistical decisions of use of the 75^th^ percentile as a cut off for good and bad working conditions is needed. Why dichotomise working conditions in this way?
