# Supplementary material for: Working conditions of the clinical health workforce in the public health facilities in Bangladesh
Source: PLoS One. 2023 Nov 20;18(11):e0294224. doi: 10.1371/journal.pone.0294224 (PMC10659196; doi:10.1371/journal.pone.0294224)
Supplement: Attachment — Submitted filename: Response to Reviewers.docx [file pone.0294224.s004.docx]

Humayun Kabir

Academic Editor

PLOS ONE

Subject: Rebuttal Letter and Revised Manuscript Submission

Dear Dr. Kabir,

We would like to express our sincere gratitude to you and the reviewers for their valuable time and insightful feedback again on our manuscript titled **Working conditions of the clinical health workforce in the public health facilities in Bangladesh**. We appreciate the constructive comments and suggestions, which have greatly contributed to the improvement of our study. In response to the reviewers' comments, we have carefully revised the manuscript, and we submit the second revised version along with this rebuttal letter.

**Reviewer #1**

**Comment:** I am sorry to obtuse, and I have carefully read the authors’ responses to Reviewer 2. As a reader of the manuscript, it is still difficult to understand how the mean weight score is calculated - I think it is across all respondents for each individual item? This seems clearer in the authors’ response.

**Response**: We have carefully reviewed your comments and made the necessary revisions in the manuscript to address this concern. In response to your comment, we revised the manuscript by incorporating the following explanation “We also calculated the mean weight score of the different components of working conditions to assess and rank their relative importance to delivering quality healthcare. In order to calculate the mean weight score, we assigned a 100-point scale (weight) to each component. The respondents were asked to rank the perceived importance of these components in delivering quality healthcare, with higher scores indicating greater importance. We obtained the individual weight scores assigned by each respondent for a particular component and then calculated the average of these scores across all respondents”.

We think, this allowed us to determine the overall mean weight score for each component. By calculating the mean weight score, we were able to assess and rank the relative importance of the different components of working conditions as perceived by the clinical workforce in delivering quality healthcare.

**Comment**: The application of the 75th percentile cut off also lacks clarity and seems where further justification or at least explanation of what it means in practice is warranted. 75th percentile of all responses? Why would this distinguish good from bad conditions?

**Response:** We appreciate the reviewer's comment and acknowledge the need for further clarification regarding the application of the 75th percentile cut-off points in categorizing the working conditions.

To provide a better understanding, we used the 75th percentile of the responses obtained from the participants as a cut-off point to categorize the working conditions as either "good" or "poor". This approach allowed us to differentiate between respondents who reported relatively higher scores (above the 75th percentile) and those who reported relatively lower scores (below the 75th percentile) on the indicators of working conditions. While we acknowledge that the interpretation of "good" and "poor" conditions may vary, the use of the 75th percentile as a cut-off point was based on the distribution of responses among the study participants. By selecting the 75th percentile, we aimed to capture the upper quartile of responses, which represented a relatively more positive assessment of working conditions.

However, we understand that further justification or clarification of the practical implications of this cut-off point is necessary. In our revised manuscript, we have provided a more detailed explanation of the rationale behind selecting the 75th percentile cut-off point and its implications for categorizing working conditions as either "good" or "poor" incorporating the following explanation “The 75th percentile cut-off point is used to distinguish between higher and lower assessments of working conditions among the study participants. It represents a threshold that separates the upper quartile of responses from the lower three quartiles. By applying the 75th percentile cut-off, we aimed to identify and categorize respondents who reported relatively more positive assessments of working conditions as "good" conditions, while those who reported relatively lower assessments were categorized as "poor" conditions.”

**Comment:** Overall, the English is good but there are occasional errors. The paper is curiously written, in future tense, as if the authors are reporting a protocol rather than on a study that has been completed. In fact, it is not even really in future tense e.g. (in the abstract) “The study aims… (rather than aimed) and Descriptive statistics, and “bivariate analysis are used to analyze the data”(rather than Descriptive statistics and bivariate analyses were used to analyze the data.” Perhaps it is because this manuscript is part of a larger study that still is underway and will be reported. This could be a discretionary revision as it does not undermine the scientific reporting although it is not following conventional reporting style.

**Response:** We have carefully reviewed your comments and made the necessary revisions throughout the manuscript to address this concern by replacing the future and present tense structure into past tense structure. However, for your clarity, this study is not a part of larger study, we just reported the results in future tense structure mistakenly.

 **Reviewer #2**

**Comment:** Abstract:
- Provide figures (numbers) in the result section
- MSR (should be mentioned)
- Remove and so on??? Line 52

**Response:** We appreciate your valuable feedback and comments on our manuscript. We have revised the manuscript accordingly.

**Comment**: Introduction section: Line 115: the study will examine remove will

**Response:** Removed accordingly.

**Comment:** In the sampling methods: How did reach that 350 providers will ensure representativeness (how did you calculate the sample size--- parameters used and formula and software etc.,)

**Response:** Thank you for your valuable feedback and comments on our manuscript. We greatly appreciate your insights.

In our study, our primary objective was to ensure the representativeness of the sample by employing a systematic sampling method. This method involved creating a comprehensive list of all clinical staff working in the selected healthcare facilities. We then used this list to randomly select respondents for participation in the study. By employing a systematic sampling approach, we aimed to achieve a structured and unbiased selection process, which is crucial for obtaining a representative sample.

Regarding the determination of the sample size, we took into account the available resources and feasibility constraints specific to our study. Initially, we aimed to interview 350 clinical workforces as an indicative sample. This sample size determination was made considering various factors, including the time, budget, and practical feasibility of conducting interviews with clinical workforces in the context of Bangladesh.

It is important to note that conducting interviews with healthcare providers in our country setting can be challenging due to limited access and time constraints. Physicians and other clinical staff often have demanding schedules and limited availability for research interviews. Additionally, public health facilities often experience a high flow of patients, further reducing the time healthcare providers can allocate for interviews. Given these practical limitations, we opted for an indicative sample size that was feasible to conduct within the available resources and time frame.

While we acknowledge that using formal statistical parameters or formulas for sample size calculation is a standard practice, it would have resulted in a larger required sample size that may have been difficult to achieve in our specific research context. Therefore, we made a practical decision based on the available resources and the need to conduct interviews with a sufficient number of participants to obtain meaningful insights.

We appreciate your suggestion to provide more clarity on the representativeness of the sample and the sample size determination. In our revised manuscript, we have provided a more detailed explanation of the factors and considerations that influenced our decision-making process in determining the sample size, including the challenges associated with conducting interviews in the healthcare setting in Bangladesh.

**Comment:** Line 149: remove (DCIs)

Response: Removed.

**Comment**: Line 163: we piloted instead of we pilot

**Response:** Revised accordingly.

**Comment:** Line 181: the merging of disagree and strongly disagree with neutral option is not logic and the rationale is not convincing.

**Response:** We appreciate the opportunity to clarify the rationale behind merging the neutral option with the 'disagree' and 'strongly disagree' options in our study.

The decision to merge these response categories was based on the specific context and purpose of our research. In the assessment of working conditions, we aimed to capture not only the positive responses but also the negative ones. By merging the neutral option with the 'disagree' and 'strongly disagree' options, we intended to account for respondents who may have selected the neutral option due to indifference or lack of positivity toward the statement in question.

Our rationale for this approach was to ensure a more comprehensive and nuanced understanding of the perceived working conditions among the respondents. By combining the negative responses, we aimed to capture a broader range of viewpoints, including those who expressed a lack of agreement or dissatisfaction with the working conditions. However, we acknowledge the reviewer's concern regarding the logic and rationale behind this approach. Upon reflection, we recognize that our justification may not have been sufficiently convincing or well-supported. We apologize for any confusion or ambiguity caused by our explanation.

**Comment:** All Tables: mention abbreviations at footnote.

**Response:** Mentioned accordingly.

**Comment**: Line 286: remove “and son on”
**Response:** Removed.

**Comment:** Line 213: abbreviations used?
**Response:** Yes, because we stated elaboration or full form of the terms/words in the previous section (sampling methods).

**Comment**: For confirmatory factor analysis: which factors clearly identified your Priori Construct?

**Response**: In our CFA model, we specified the a priori construct of "working conditions" and expected the observed indicators to load strongly on this construct. We evaluated the factor loadings, which indicate the strength of the relationship between each indicator and the latent construct. Our criteria for determining the clear identification of factors were factor loadings of 0.50 or higher, indicating a substantial correlation between the indicator and the latent construct. Based on the results of our CFA, we found that most indicators had substantial factor loadings of 0.50 or higher, indicating a strong correlation with the latent construct of working conditions. For example, functional medical equipment, provider’s safety, stress-free workplace, recognition of work, clean workplace, color and lighting condition, ventilation, aesthetic workplace, washroom facilities, uninterrupted power supply, uninterrupted water supply, damp-free workplace, respect to opinions, valuation of work, mentoring, and medicine and MSR supply all these factors clearly identified our Priori construct.

**Comment:** Provide goodness of fit statistics for the Table 7.

**Response:** Thank you for your comment regarding the inclusion of goodness of fit statistics in Table 7. We appreciate your suggestion, but we have made a deliberate decision not to provide goodness of fit statistics in this particular table.

Table 7 in our manuscript focuses specifically on the psychometric analysis results for the indicators of working conditions. It provides important information such as factor loadings, uniqueness values, inter-item correlations, and Cronbach's alpha values. These metrics are essential for evaluating the reliability and validity of the measurement model. While goodness of fit statistics, such as chi-square, RMSEA, CFI, and SRMR, are commonly used to assess the overall fit of a model in confirmatory factor analysis, they are not directly related to the psychometric properties of individual indicators. Therefore, considering the specific focus of Table 7 on the psychometric properties of the individual indicators, we believe it is more appropriate to exclude the goodness of fit statistics in this table. This approach allows for a clearer and more focused presentation of the results related to the psychometric analysis of the indicators.

**Comment**: From line 319 to line 322: repetition and not need in the outset of the discussion section

**Response**: Accepted, and we deleted the lines 319 to line 322 from the discussion section.

**Reviewer#3**

**Comment:** I thank the authors for updating the manuscript. My comment has been fully addressed in the revised version.

**Response**: Thank you for reviewing the revised version of our manuscript. We are pleased to hear that your comment has been fully addressed and that you are satisfied with the updates we made. We appreciate your time and valuable feedback, which has helped us improve the quality of our work.

**Reviewer#5**

**Comment:** From the revised manuscript and authors' response to the comments, it seems that the authors have made their best attempt to improve the manuscript as per all reviewers’ comments and feedback. However, I think that the manuscript still is not in good shape, especially the statistical analysis part. This study is based on inappropriate statistical analysis with improper interpretation.

**Response:** Thank you for your feedback on the revised version of our manuscript. We respect your perspective and concerns regarding the statistical analysis and interpretation. We have carefully considered your comment and understand your viewpoint. We would like to assure you that we have taken great care in conducting the statistical analysis and interpreting the results. We have followed standard procedures and guidelines for data analysis and have made efforts to provide clear and accurate interpretations of our findings.

We value your expertise and insights, and we are committed to producing a high-quality manuscript. We are open to your suggestions and will carefully consider any further feedback you provide. Thank you for your attention and assistance in improving the quality of our work.

However, we would like to provide some rationale as to why we believe the present format of the statistical analysis is appropriate for our study.

Methodological appropriateness: The statistical analysis conducted in our study was chosen based on the research question, study design, and data characteristics. We carefully selected appropriate statistical tests and methods that align with the objectives of our study and the nature of our data. We followed established guidelines and best practices in the field to ensure methodological rigor.

Transparency and reproducibility: We have provided detailed descriptions of the statistical procedures and methods employed in the manuscript. This allows readers and future researchers to understand and replicate our analysis. We have also included references to the appropriate statistical literature and resources to support our choices.

Statistical significance: We have reported statistical significance in our analysis. Statistical significance helps determine whether the observed results are likely to have occurred by chance. By including this measure, we provide a comprehensive understanding of the results.

Interpretation within context: We have taken into account the limitations and context of our study when interpreting the statistical results. We have considered factors such as sample size, variability, and potential confounders in our analysis and interpretation. We have also discussed the implications and relevance of our findings in relation to the existing literature and theoretical frameworks.

While we acknowledge that there is always room for improvement and alternative approaches, we believe that the present format of the statistical analysis is appropriate for our study given its objectives and constraints. We remain open to constructive feedback and suggestions for improvement, and we are committed to addressing any specific concerns you may have to enhance the quality and validity of our work.

**Editorial Comment**

Journal Requirement

Please review your reference list to ensure that it is complete and correct. If you have cited papers that have been retracted, please include the rationale for doing so in the manuscript text, or remove these references and replace them with relevant current references. Any changes to the reference list should be mentioned in the rebuttal letter that accompanies your revised manuscript. If you need to cite a retracted article, indicate the article’s retracted status in the References list and also include a citation and full reference for the retraction notice.

**Response:** We have carefully reviewed our reference list again. We have not cited any papers that have been retracted.

We sincerely hope that these revisions address the concerns raised by the reviewers. We are confident that the revised manuscript represents a significant improvement and contributes to the existing literature on working conditions of clinical health workforces. We once again express our appreciation for the opportunity to submit our work to PLOS ONE.

Thank you for considering our revised manuscript and rebuttal letter. We look forward to hearing from you regarding the final decision.

Sincerely,

Md. Ragaul Azim

Corresponding Author

Institute of Health Economics, University of Dhaka

Email: [azim.ihe@du.ac.bd](mailto:azim.ihe@du.ac.bd)
