# Supplementary material for: Working conditions of the clinical health workforce in the public health facilities in Bangladesh
Source: PLoS One. 2023 Nov 20;18(11):e0294224. doi: 10.1371/journal.pone.0294224 (PMC10659196; doi:10.1371/journal.pone.0294224)
Supplement: Attachment — Submitted filename: Response to Reviewers.docx [file pone.0294224.s005.docx]

Humayun Kabir

Academic Editor

PLOS ONE

Subject: Rebuttal Letter and Revised Manuscript Submission

Dear Dr. Kabir,

We greatly appreciate the time and effort that you and the esteemed reviewers have dedicated to assessing our manuscript titled "**Working conditions of the clinical health workforce in the public health facilities in Bangladesh**". We are writing to express our gratitude for the opportunity to respond to the reviewers' comments and to outline the revisions that we have made based on their valuable feedback.

In the third round of reviews, we were pleased to see that two out of the four reviewers acknowledged our efforts in addressing their concerns and improving the manuscript. We made every effort to carefully consider and incorporate their suggestions, which we believe has significantly strengthened the quality and rigor of our study. For the issues raised by the remaining two reviewers, we diligently revised the manuscript in accordance with their feedback. We addressed both the additional concerns and the issues that were previously raised. We submit the third revised version along with this rebuttal letter.

**Reviewer #1**

**Comment:** The authors have responded appropriately to my previous concerns and to those of other reviewers. I can now follow the methods, and although I have reservations as to whether the 75th percentile is an appropriate way to separate good from bad working conditions (this is just a relative measure, rather than against some set of standards), at least the authors have described their methods and rationale.

**Response:** Thank you for taking the time to review our manuscript once again and for recognizing our efforts in addressing your concerns as well as those of other reviewers. We understand your reservations about using the 75th percentile as a criterion for categorizing working conditions and appreciate your feedback on this matter. We agree that this approach is relative and not based on specific standards. Our intention was to establish a differentiation point within the data for the purpose of our analysis. We have described our methods and rationale thoroughly to provide transparency in our approach. Your feedback was invaluable in enhancing the quality of our manuscript.

**Reviewer #2**

**Comment:** How the sample size was calculated?

**Response:** We responded to this question previously in the last paragraph of the sampling methods section. Basically, we employed an indicative sampling to determine the sample size. We stated this in our manuscript as follows “To ensure the representativeness of the sample, the study utilized a systematic sampling method, which involved using a list of all clinical staff in the selected healthcare facilities for the randomization of the respondents. At the outset, we contacted 350 clinical workforces for interviews, consisting of 120 physicians and 230 other clinical staff, to serve as an indicative sample. Regrettably, 31 of them declined to participate. Consequently, we conducted interviews with 319 clinical workforces, comprising 109 physicians and 210 other clinical staff. This sample size determination was made considering various factors, including the time, budget, and practical feasibility of conducting interviews with clinical workforces in the context of Bangladesh. Physicians and other clinical staff often have demanding schedules and limited availability for research interviews. Additionally, public health facilities often experience a high flow of patients, further reducing the time healthcare providers can allocate for interviews. Given these practical limitations, we opted for an indicative sample size that was feasible to conduct within the available resources and timeframe.”.

**Comment:** How participants form each unit were recruited?

**Response:** Thank you for your question. We appreciate your attention to this aspect of our study. In our research, we didn't recruit participants based on units. Instead, we employed a systematic sampling method to ensure the representativeness of the sample. This method involved utilizing a list of all clinical staff in the selected healthcare facilities, not based on separate units and then randomly selecting respondents from this list. We have outlined this process in the methodology section of our manuscript to provide clarity on our approach in the last part of the sampling methods selection.

**Comment:** Why the weight mean for each response was calculated?

**Response:** Thank you for your question regarding the calculation of the weighted mean for each response in our data analysis. The purpose of computing the weighted mean was to account for the varying significance that clinical workforces might assign to different components when assessing their working conditions. This approach allowed us to give appropriate weightage to the responses, reflecting their relative importance in contributing to the overall status of working conditions. We aimed to provide a more accurate and comprehensive evaluation by considering both the respondents' scores for each component and their individual significance. This approach is explained in the data analysis section of the revised manuscript.

**Comment:** The authors overstepping the value of their sample size to represent geography and other variables which in not the case actually.
**Response:** We appreciate the reviewer's feedback and would like to clarify our intent regarding the representation of geography and other variables. Our study aimed to achieve a sample that represents a diverse cross-section of the clinical workforce in the selected healthcare facilities. While we acknowledge that our sample might not comprehensively capture all geographic and other variations, we strived to ensure a balanced representation of different backgrounds within the constraints of our resources and study scope.

**Comment:** What were the results of the pilot testing and how many providers were used to carry out this pilot testing?

**Response:** During the initial phase of the study, we conducted a pilot testing of the questionnaire to assess its reliability and validity within the country context. The pilot involved a sample of 30 clinical providers from various healthcare facilities resembling our study population. The primary objective of the pilot was to evaluate the clarity, coherence, and appropriateness of the questionnaire items for our target respondents. While we didn't explicitly report the pilot results in the manuscript, we utilized the feedback received from the pilot participants to refine the questionnaire's design. This iterative process aided in enhancing the questionnaire's relevance and suitability for our study population. We acknowledge the reviewer's suggestion and have incorporated a concise description of the pilot process, including the number of participants involved, to provide further transparency in our Data Collection Instrument section of the methodology.

**Comment:** What are the possible predictors and (confounders) if possible? How the authors find those factors responsible for the participant responses?

**Response:** We appreciate the reviewer's interest in exploring possible predictors and confounders in our study. However, our study primarily focused on assessing the working conditions of healthcare providers. While we did not explicitly collect data on potential predictors or confounding factors, we acknowledge the importance of such analyses for a more comprehensive understanding of the relationships involved. Given the scope and objectives of our study, we did not incorporate these aspects into our research design. Nonetheless, we value the suggestion and will consider incorporating relevant analyses in future studies to explore potential predictors and confounders related to participant responses.

**Comment:** The conduction of confirmatory factor analysis may be misleading considering this small sample size (factor analysis is not the sole items to determine validity), the construct validity is more important to be assessed in this case.

**Response:** We appreciate the reviewer's concern about the use of confirmatory factor analysis (CFA) in relation to our sample size. We acknowledge the importance of construct validity assessment, especially in the context of a smaller sample size. While CFA is a method, we employed to assess the construct validity of our survey tools, we understand that the reliability of this analysis can be affected by sample size limitations. In hindsight, considering the smaller sample size, employing alternative methods could indeed provide a more comprehensive view of construct validity. In our revised manuscript both in the data analysis section and limitations section, we have provided a balanced discussion of the limitations and potential implications of our chosen psychometric analysis approach, particularly in light of the sample size. This will allow readers to better understand the context and potential constraints of our construct validity assessment.

**Comment**: The introduction section is so redundant, with many sentences describing the aims and objectives of the study.

**Response:** We appreciate your thorough review. We understand your concern about the redundancy in the introduction section. We have reviewed the section carefully and ensured that the aims and objectives of the study are stated concisely and without repetition.

**Comment**: The introduction section should emphasize the importance of the study within the national context more clearly rather than the international.

**Response**: We appreciate your observation. We have reviewed the section carefully and ensured that our manuscript has already aligned with your suggestion. We have focused on emphasizing the significance of the study within the national context rather than emphasizing the international context. Your input has been valuable in shaping our approach to the introduction section.

**Comment**: You can’t provide concrete evidence form a cross-sectional study based on self-reporting questions (consider this while providing your introduction and in the discussion section.
**Response:** We acknowledge the inherent limitations of cross-sectional studies based on self-reporting questions. In our limitations section, have provided a balanced perspective on the limitations of our methodology and the potential impact on the robustness of our findings.

**Comment:** Both the introduction and discussion sections are too lengthy and it need to be shortened and concise and to the point.

**Response:**

About Introduction section: We acknowledge that the introduction section has become more extensive due to the incorporation of relevant issues based on feedback from other reviewers in previous rounds. We believe that these additions contribute to the comprehensive understanding of the research context and significance.

About Discussion section: While we acknowledge that the discussion section is large and comprehensive due to the inclusion of similarities and dissimilarities of other studies along with their implications, we believe this approach is crucial to provide a well-rounded context for our findings. We ensured that the section remains concise and focused on key points while retaining its relevance and depth. Your suggestions are valuable in ensuring the clarity and effectiveness of our manuscript.

**Reviewer #3**

**Comment:** My comments have already been addressed in the previous round, and I commend the authors for further enhancing the manuscript based on additional reviewers' inputs. The manuscript has indeed been improved, effectively addressing the issues raised.

**Response**: Thank you very much for your thoughtful assessment of our manuscript and your positive feedback on the revisions made. We are pleased to hear that the changes we implemented based on your and other reviewers' feedback have contributed to enhancing the quality of the manuscript. Your guidance has been immensely valuable throughout this process, and we are grateful for your continued engagement.

**Reviewer #5**

Comment: I couldn't see any changes in the statistical analysis part. If the authors are unwilling to make any changes, it's OK but at least change the tables the way it is presented. The tables should be presented in an intelligent way. The tables should be self-explanatory. In the present form, it is impossible to read and understand the tables and the interpretation.

**Response**: Thank you for your valuable feedback on our manuscript. We deeply appreciate your insights and suggestions regarding the presentation of the statistical analysis results, particularly in the context of the extensive assessment components in our study. We acknowledge your concern about the length and complexity of the tables. We agree that clarity and readability are paramount in presenting our findings. Though, we have made every effort to present the data in a more organized and intelligible manner, while also adhering to the limitations imposed by the volume of information we needed to convey, we have no way to present the data otherwise. In response to your comment, we have reviewed the structure of the tables, and we still believe in the current format they are self-explanatory.

We sincerely hope that these revisions address the concerns raised by the reviewers. We are confident that the revised manuscript represents a significant improvement and contributes to the existing literature on working conditions of clinical health workforces. We once again express our appreciation for the opportunity to submit our work to PLOS ONE.

Thank you for considering our revised manuscript and rebuttal letter. We look forward to hearing from you regarding the final decision.

Sincerely,

Md. Ragaul Azim

Corresponding Author

Institute of Health Economics, University of Dhaka

Email: [azim.ihe@du.ac.bd](mailto:azim.ihe@du.ac.bd)
