# Supplementary material for: Working conditions of the clinical health workforce in the public health facilities in Bangladesh
Source: PLoS One. 2023 Nov 20;18(11):e0294224. doi: 10.1371/journal.pone.0294224 (PMC10659196; doi:10.1371/journal.pone.0294224)
Supplement: Attachment — Submitted filename: Response to Reviewers.docx [file pone.0294224.s006.docx]

Academic Editor

PLOS ONE

Subject: Rebuttal Letter and Revised Manuscript Submission

Dear Editor,

We greatly appreciate the time and effort that you and the esteemed reviewers have dedicated to assessing our manuscript titled "**Working conditions of the clinical health workforce in the public health facilities in Bangladesh**". We are writing to express our gratitude for the opportunity to respond to the reviewers' comments and to outline the revisions that we have made based on their valuable feedback.

In the fourth round of reviews, we are appreciative of the constructive feedback provided by the reviewers. It is gratifying to note that two out of the three reviewers have acknowledged our diligent efforts in addressing their concerns and enhancing the manuscript. We have taken great care to thoughtfully consider and implement their valuable suggestions, resulting in substantial improvements in the quality and rigor of our study.

However, it is essential to emphasize that, from our perspective, the manuscript has been meticulously revised, and we believe that it has reached a level of quality and completeness that aligns with the objectives and scope of the journal. We have diligently addressed all the comments and concerns raised throughout the review process.

Therefore, at this juncture, we respectfully request the editor's kind discretion regarding the comments, our responses, and the decision on whether the manuscript should proceed further. We believe that the manuscript is now well-prepared for the next steps in the publication process.

However, we submit the fourth revised version along with this rebuttal letter.

**Reviewer #1**

**Comment:** I would suggest that Table 6 is does not express the mean weights to 2 decimal places. This just makes the table less busy and easier to read but does not change the data or its interpretation.

**Response:** Thank you for your valuable feedback and suggestion regarding Table 6 in our manuscript. We agree that expressing the mean weights to one decimal place, rather than two, makes the table less cluttered and more reader-friendly while retaining the integrity of the data and its interpretation. We have implemented this change by reducing the number of digits after the decimal point in Table 6 accordingly.

**Reviewer #2**

**Comment:** Abstract

- numbers in the abstract examples 3.4, 99.15, should be corrected,

- P less or equal to 0.05 - should be corrected

**Response:** Regarding your first comment about the numbers in the abstract (3.4 and 99.15), we have rechecked our data, and we can confirm that these values are accurate.

Concerning your second comment about the presentation formatting of the p-value, we acknowledge this and agree that it should be corrected to align with accepted academic standards. We have made the necessary adjustments to ensure that the p-value is properly formatted as "p ≤ 0.05." Once again, we appreciate your time and effort in reviewing our manuscript, and we will promptly implement these corrections to improve the accuracy and clarity of our work.

We sincerely hope that these revisions address the concerns raised by the reviewers. We are confident that the revised manuscript represents a significant improvement and contributes to the existing literature on working conditions of clinical health workforces. We once again express our appreciation for the opportunity to submit our work to PLOS ONE.

Thank you for considering our revised manuscript and rebuttal letter. We look forward to hearing from you regarding the final decision.

Sincerely,

Md. Ragaul Azim

Corresponding Author

Institute of Health Economics, University of Dhaka

Email: [azim.ihe@du.ac.bd](mailto:azim.ihe@du.ac.bd)
