# Supplementary material for: Working conditions of the clinical health workforce in the public health facilities in Bangladesh
Source: PLoS One. 2023 Nov 20;18(11):e0294224. doi: 10.1371/journal.pone.0294224 (PMC10659196; doi:10.1371/journal.pone.0294224)
Supplement: Attachment — Submitted filename: Response to Reviewers.docx [file pone.0294224.s007.docx]

Academic Editor

PLOS ONE

Subject: Rebuttal Letter and Revised Manuscript Submission

Dear Editor,

We greatly appreciate the time and effort that you and the esteemed reviewers have dedicated to assessing our manuscript titled "**Working conditions of the clinical health workforce in the public health facilities in Bangladesh**". We are writing to express our gratitude for the opportunity to respond to the reviewers' comments and to outline the revisions that we have made based on their valuable feedback.

In the 5^th^ round of reviews, we are appreciative of the constructive feedback provided by the reviewers. We have taken great care to thoughtfully consider and implement their valuable suggestions, resulting in substantial improvements in the quality and rigor of our study.

However, it is essential to emphasize that, from our perspective, the manuscript has been meticulously revised, and we believe that it has reached a level of quality and completeness that aligns with the objectives and scope of the journal. We have diligently addressed all the comments and concerns raised throughout the review process. **We would not be eager to respond to any further comments by the reviewers as it already has taken long time in the review process and we are responding to 5^th^ review this time.**

Therefore, at this juncture, we respectfully request the editor's kind discretion regarding the comments, our responses, and the decision on whether the manuscript should proceed further. We believe that the manuscript is now well-prepared for the next steps in the publication process.

However, we submit the fifth revised version along with this rebuttal letter.

**Reviewer #2**

**Comment:** There is limited research on measuring the status of working conditions of clinical health workers in both national and international contexts. As evidenced above, some studies have focused on specific aspects of health workers' working conditions in Bangladesh, such as job satisfaction or motivation. However, a comprehensive analysis of the various dimensions of health workers' working conditions is lacking. Such an analysis is critical in understanding the challenges and opportunities in improving the working conditions and consequently, the quality of care provided to the population”
This section needs correction:
- Remove “evidence” and replace it with other appropriate word
- Lacking of reference all through.
- However----- (very long sentence).

**Response:** Revised accordingly.

To address the first comment, we have replaced “evidence” with “stated”.

About second comment of this section: As evidenced above, some studies have focused on specific aspects of health workers' working conditions in Bangladesh, such as job satisfaction or motivation…. For this sentence, all the references have been mentioned in the previous section separately, as this statement is a linking statement of the previous section pointing out literature review. References 3, 5, 6,8, 9,10,11 are the examples for your kind attention.

About third comment of this section: Revised as suggested.

**Comment:** The current study aims to fill this knowledge gap by providing a comprehensive analysis of the working conditions of clinical health workforce in public health facilities in Bangladesh. The study examined the level of various components of working conditions, including their workload, remuneration, training, workplace layout, safety, water supply, job recognition, and other support systems. This study also assessed the relative importance of the different factors of working conditions to deliver quality care. The findings of the study will be of interest to policymakers, healthcare managers, and researchers, both in

- This section can be summarized in few sentences and please do not exaggerate the outcomes of your paper and stick to one major objective.

**Response**: Revised as suggested.

**Comment:** In all tables: mention the test of significance in the footnote.
**Response:** Mentioned accordingly in the table 4 and 5, where there is stated P-value

**Comment**: In all tables first mention the number (%) and not %(no.)

**Response:** Revised accordingly where appropriate (Table 4 and 5).

**Comment**: Table 6: abbreviations at the footnote????

**Response:** Added footnote as suggested.

**Comment:** Table 7: why reporting interitem correlation coefficients?

**Response**: We have reported interitem correlation for the following reasons:

1. Data Transparency: It provides transparency about how closely related or unrelated individual items are within a scale. This transparency is essential for readers to understand the structure and reliability of the measurement instrument.
2. Assessing Internal Consistency: Interitem correlation coefficients help assess the internal consistency of a scale or construct. In our study, we reported these to demonstrate how well the items within the construct are related to each other.
3. Dimensionality and Validity: By examining interitem correlations, readers of the study can explore the dimensionality of a construct and assess the validity of the measurement tool.

**Comment:** According to the cutoff used, several items of the factor loading are not in conformity with the construct of the data collection tool???? Explain, despite mentioning it in the limitation section this point would compromise the internal validity of your study?????

**Response:** We sincerely appreciate your insightful feedback regarding the observed discrepancies in items not fully conforming to the construct of our data collection tool and the potential implications on the internal validity of our study. While we acknowledge the potential impact of non-conforming items on the internal validity of our study, we believe that our detailed discussions, rigorous data analysis, factor loadings, uniqueness values, and Cronbach alpha coefficients assessments have allowed us to make valid inferences within the boundaries of this limitation.

We sincerely hope that these revisions address the concerns raised by the reviewer. We are confident that the revised manuscript represents a significant improvement and contributes to the existing literature on working conditions of clinical health workforces. We once again express our appreciation for the opportunity to submit our work to PLOS ONE.

Thank you for considering our revised manuscript and rebuttal letter. We look forward to hearing from you regarding the final decision.

Sincerely,

Md. Ragaul Azim

Corresponding Author

Institute of Health Economics, University of Dhaka

Email: [azim.ihe@du.ac.bd](mailto:azim.ihe@du.ac.bd)
